# A Novel Loop-Mediated Isothermal Amplification Assay for Rapid Detection of *Yersinia pestis*

**DOI:** 10.3389/fmicb.2022.863142

**Published:** 2022-04-07

**Authors:** Ying Bai, Maria Rosales Rizzo, Christina Parise, Sarah Maes, Rebecca J. Eisen

**Affiliations:** Bacterial Diseases Branch, Division of Vector-Borne Diseases, Centers for Disease Control and Prevention, Fort Collins, CO, United States

**Keywords:** assay development, LAMP, dry bath heater, real-time LAMP, plague, *Yersinia pestis*

## Abstract

Rapid detection of *Yersinia pestis*, the causative agent of plague, is essential during field investigations to enable prompt control measures for prevention of the spread of the disease. Affordable, efficient, reliable, and simple detection assays are extremely useful, particularly in plague-endemic regions with limited resources. We developed a loop-mediated isothermal amplification (LAMP) assay that detects *Y. pestis* within 30 min by simply incubating at 65°C on a dry bath heater. The assay targeted the *caf1A* gene that is situated on the pMT1 plasmid using six specific primers. *Y. pestis* presence is visually detected based on the color change in the reactions. For comparison of the assay performance, a real-time LAMP with fluorescent dye detection was conducted on a real-time PCR instrument using the same six primers. Sensitivity assessment showed that the limit of detection (LOD) was 0.2 and 0.03 pg when performed on the dry bath heater and on the real-time PCR instrument, respectively. The assay was 100% specific, having no cross-reactivity with closely related *Yersinia* spp. and other bacterial species. We tested the LAMP assay on field-collected fleas and showed that it successfully detected *Y. pestis* with identical results to that of a previously published pentaplex real-time PCR assay. These findings suggest that the relatively inexpensive and simpler LAMP assay could be used to support field investigations, yielding comparable results to more expensive and complex PCR assays.

## Introduction

*Yersinia pestis*, classified as a category “A” biothreat agent, is the bacterial causative pathogen of plague, a deadly infectious zoonotic disease. The bacterium mainly infects rodents that are its natural reservoirs and can be transmitted to humans and some other mammals through infected rodent flea bites (Stenseth et al., 2008). Plague outbreaks often occur in geographic regions that lack basic laboratory infrastructure for diagnostics (Butler, 2013; Grácio and Grácio, 2017). Due to the highly infectious nature and mortality rate of plague, accurate and rapid direct detection of *Y. pestis* in the early stages of epizootics is critical for containment of the plague outbreak as well as for environmental and medical management. Several surveillance and response strategies have been proposed to identify where and when plague epizootics are occurring, with the intent of directing interventions to prevent human plague cases (Gage, 1999). These strategies include monitoring plague host abundance, host mortality (die-offs or rat falls), flea loads on rodents, seropositivity of non-human hosts, or direct detection of *Y. pestis* in vectors or rodent hosts (Borchert et al., 2012; Moore et al., 2015; Boegler et al., 2018; Ehlers et al., 2020). The most effective and reliable strategies are dependent on accurate detection of *Y. pestis* (Eisen et al., 2020, 2021).

Multiple methods have been employed for plague diagnosis. Bacterial culture is the gold standard for plague diagnosis in humans, vectors, and non-human hosts; however, this procedure is time-consuming and poses biosafety risks to laboratory staff, with strict requirements of performance in biosafety level 3 laboratories (Norkina et al., 1994). Serology is mainly used in late stages of the infection for detection of plague antibodies (Splettstoesser et al., 2004; Simon et al., 2013; Hsu et al., 2018). While useful for identifying evidence of past plague activity, serology is not suitable for investigating plague in rodents due to the extremely high mortality (Enscore et al., 2020). In recent years, polymerase chain reaction (PCR) and other molecular diagnostic techniques have been developed and largely applied for *Y. pestis* detection (Campbell et al., 1993; Neubauer et al., 2000; Tomaso et al., 2003; Stewart et al., 2008; Bai et al., 2020). However, the requirement for a high level of technical expertise and high-precision thermal cyclers for amplification may limit this powerful technology from being widely used in areas of greatest need.

Loop-mediated isothermal amplification (LAMP) is a relatively new molecular technique that amplifies nucleic acid under isothermal conditions (Notomi et al., 2000). Using a DNA polymerase with high displacement strand activity and a set of four to six specific primers, LAMP amplifies DNA with high sensitivity, specificity, and rapidity. In contrast to PCR, LAMP does not require thermal cyclers. A simple dry/water bath that provides a constant temperature will fulfill the amplification. Furthermore, because a pH indicator dye was used in LAMP reactions, the amplified products can be visually detected based on the color change in the reaction (Pal et al., 2018). These features make LAMP an excellent application for pathogen detection, especially for use in resource-limited areas in developing countries. LAMP techniques are increasingly used in molecular diagnostics as a potential alternative to PCR-based methods (Pal et al., 2018; Bodulev and Sakharov, 2020; Mukama et al., 2020) and already have been successfully applied for detection of bacteria, fungi, parasites, and viruses (Kuboki et al., 2003; Gonçalves et al., 2014; Lass et al., 2017; Lu et al., 2020). Several LAMP assays for *Y. pestis* detection have been developed as well (Nunes et al., 2014; Feng et al., 2018; Randriantseheno et al., 2020; Singh et al., 2020).

During the evolutionary history of *Y. pestis*, the bacterium acquired two unique plasmids, pMT1 and pPCP1, in addition to pCD1 that is shared with *Yersinia pseudotuberculosis* and *Yersinia enterocolitis* (Ben-Gurion and Shafferman, 1981; Ferber and Brubaker, 1981). Nevertheless, not all three plasmids are present in all *Y. pestis* strains (Butler, 1972; Filippov et al., 1990; Meka-Mechenko, 2003). Previously developed *Y. pestis* LAMP assays have mainly used *caf1* (Nunes et al., 2014; Randriantseheno et al., 2020; Singh et al., 2020), a highly specific gene that is situated in the pMT1 plasmid and plays an essential role for *Y. pestis* virulence by expressing the capsular antigen fraction 1 (Caf1) protein (Sebbane et al., 2009; Al-Jawdah et al., 2019). However, paired loop primers seemed difficult to generate when using the *caf1* gene. There was only one loop primer included in previous assays (Nunes et al., 2014; Randriantseheno et al., 2020). In this study, we developed a new LAMP assay for rapid detection of *Y. pestis*. Our main goal was to apply the assay for testing fleas, rodents, and other environmental samples collected during field investigations and/or in plague-endemic regions for *Y. pestis* infection. When dealing with a large sample size, a simple, rapid, and efficient assay would be very helpful. Previously, Hanson et al. (2007) used a nested PCR for investigation of *Y. pestis* infection in large scale of fleas collected from prairie dog burrows. Although it worked, the nested PCR required a thermal cycler and took several hours to complete. Our LAMP assay targeted the *caf1A* gene, which is a subunit of the *caf1* operon. We (1) designed a set of six *caf1A*-specific primers, (2) optimized reaction temperature and reaction time, (3) assessed the sensitivity and specificity, (4) evaluated the performance by checking the interference of mouse DNA and testing *Y. pestis* strains with different plasmid profiles, and (5) tested the applicability by screening field-collected fleas. A dry bath heater was used to perform the LAMP assay. We also performed the assay using a real-time PCR instrument to evaluate the performance of the LAMP assay under simple and less costly isothermal conditions. The terms “standard LAMP” or “real-time LAMP” were used when the assay was performed on a dry bath heater or a real-time PCR instrument, respectively.

## Materials and Methods

### Primer Design

Employing the Primer Explorer V4 software^1^ (Eiken Chemical Co., Ltd., Tokyo, Japan) and using the sequences of *Y. pestis* EV vaccine strain (GenBank accession no. AY450845), we first designed two outer primers (F3 and B3) and two inner primers (FIP and BIP); then two loop primers (LF and LB) were generated based on the sequences of F3/B3 and FIP/BIP. Together, a set of six LAMP primers were designed (Table 1). The screening parameters included in primer selection were the GC (guanine–cytosine) content, Tm (melting temperature), stability of the primer sequence, secondary structure formation, and distance between primers, which were assessed to determine the final primer sequences.

**TABLE 1:** Nucleotide sequences of the LAMP *caf1*A primers.

Function	Primer name	Primer sequences (5′–3′)
Outer primer	F3	AGACAAGAAAATTCAGAGAAGG
	B3	TGTCAAAGATACTGCTATCAGAA
Inner primer	FIP	TGAACTACGAAAGCGCCAAG-AGGCAAGTCTCTGGACTC
	BIP	AGGATGGCAGCGTTCGTATA-TTCCCCCAATGTCAAACG
Loop primer	LF	TCCCGGTTGCAACTGAGCA
	LB	TGCCGAGCGAGGATTGAATACA

### DNA Template of *Yersinia pestis* and Other Bacterial Species

*Yersinia pestis* strain CO96-3188 is a fully virulent North American biovar Orientalis strain (Engelthaler et al., 2000; Eisen et al., 2006). Genomic DNA extracted from this strain was used for the assay development in this work. DNA from 11 other *Y. pestis* strains varying in plasmid profiles was included for further evaluation of the assay (Table 2). DNAs from 10 *Y. pseudotuberculosis* strains, 2 *Y. enterocolitica* strains, and 20 non-*Yersinia* strains representing a wide variety of different bacterial species including *Bacillus anthracis*, *Bartonella* spp., *Borrelia* spp., *Brucella canis*, *Burkholderia cenocepacia*, *Escherichia coli*, *Francisella tularensis*, *Leptospira interrogans*, *Listeria monocytogenes*, *Rickettsia sibirica*, *Salmonella enterica*, *Staphylococcus aureus*, *Toxoplasma gondii*, and *Trypanosoma cruzi* were included for specificity testing of the assay (Table 3). Although many of these species would not be expected in flea specimens (the sample type upon which this assay was evaluated), most could be encountered if the assay is later applied to mammalian samples. DNA templates of the above-mentioned strains used in the study were obtained from BEI Resources, University of Texas Medical Branch (UTMB), and CDC Bacterial Diseases Branch (Tables 2, 3).

**TABLE 2:** Loop-mediated isothermal amplification testing results for *Y. pestis* strains with different plasmid profiles.

ID	Strain	Source	Plasmid profile			LAMP testing	
			pMT1	pCD1	pPCP1	Dry bath incubation	Real-time LAMP
NR-2715	ZE94-2122	BEI	yes	yes	yes	pos	pos
NR-2716	PEXU2	BEI	yes	yes	yes	pos	pos
NR-2717	CO92	BEI	yes	yes	yes	pos	pos
NR-2644	A1122	BEI	yes	no	yes	pos	pos
NR-4711	TS D4	BEI	yes	no	yes	pos	pos
NR-4715	Kuma D8	BEI	yes	no	yes	pos	pos
NR-4716	Yokohama D10	BEI	yes	no	yes	pos	pos
NR-4718	Kimberley D12	BEI	yes	no	yes	pos	pos
NR-2645	KIM10 +	BEI	yes	no	no	pos	pos
NR-4712	A12 D5	BEI	yes	no	no	pos	pos
NR-2718	PB6	BEI	no	yes	no	neg	neg

*Four replicates were tested for each template; DNA concentration: 1 pg.*

**TABLE 3:** Specificity testing of the LAMP assay.

ID	Strain	Bacterial species	Source	LAMP testing	
				Dry bath incubation	Real-time LAMP
NR-4647	IP2666	*Yersinia pseudotuberculosis*	BEI	neg	neg
NR-4648	IP2515	*Yersinia pseudotuberculosis*	BEI	neg	neg
NR-4649	IP2777	*Yersinia pseudotuberculosis*	BEI	neg	neg
NR-4650	IP2790	*Yersinia pseudotuberculosis*	BEI	neg	neg
NR-4651	IP2775	*Yersinia pseudotuberculosis*	BEI	neg	neg
NR-4652	YPIII (p−)	*Yersinia pseudotuberculosis*	BEI	neg	neg
NR-4653	YPIII (p+)	*Yersinia pseudotuberculosis*	BEI	neg	neg
4284		*Yersinia pseudotuberculosis*	UTMB	neg	neg
	1A	*Yersinia pseudotuberculosis*	CDC	neg	neg
	B15	*Yersinia pseudotuberculosis*	CDC	neg	neg
NR-3064	Billups-1803-68	*Yersinia enterocolitica*	BEI	neg	neg
NR-3065	WA	*Yersinia enterocolitica*	BEI	neg	neg
NR-9544	Ty2	*Bacillus anthracis*	BEI	neg	neg
		*Bartonella doshiae*	CDC	neg	neg
		*Bartonella phoceensis*	CDC	neg	neg
		*Bartonella rochalimae*	CDC	neg	neg
		*Bartonella washoensis*	CDC	neg	neg
		*Borrelia burgdorferi*	CDC	neg	neg
		*Borrelia mayonii*	CDC	neg	neg
		*Brucella canis*	CDC	neg	neg
NR-36042	MA00-2987	*Burkholderia cenocepacia*	BEI	neg	neg
NR-2647	KM14S	*Escherichia coli*	BEI	neg	neg
NR-3020	OR96-0246	*Francisella tularensis* ssp. *holarctica*	BEI	neg	neg
NR-3028	Sterne BA781	*Francisella tularensis* ssp. *novicida*	BEI	neg	neg
NR-3017	Weybridge	*Francisella tularensis* ssp. *tularensis*	BEI	neg	neg
		*Leptospira interrogans*	CDC	neg	neg
NR-13354	TCH1516	*Listeria monocytogenes*	BEI	neg	neg
		*Rickettsia sibirica*	CDC	neg	neg
NR-543	FSL J2-064	*Salmonella enterica* ssp. *enterica*	BEI	neg	neg
NR-12252	VEG	*Staphylococcus aureus*	BEI	neg	neg
NR-33510	LMG 16656	*Toxoplasma gondii*	BEI	neg	neg
		*Trypanosoma cruzi*	CDC	neg	neg

*Four replicates were tested for each sample. DNA concentration: 100 pg.*

### Loop-Mediated Isothermal Amplification Reaction Setup

The *caf1A* primer mix (10×) was first prepared, which contained 2 μM F3 and B3 each; 16 μM FIP and BIP each; and 4 μM LF and LB each. LAMP reactions with a total volume of 25 μL were set up in two different systems depending on whether we were using the real-time PCR instrument or the dry bath heater. For the real-time LAMP, the reaction contained 12.5 μl of WarmStart LAMP 2 × Master Mix (New England Biolabs, Ipswich, MA, United States), 0.5 μl of fluorescent dye (New England Biolabs, Ipswich, MA, United States), 2.5 μl 10 × *caf1A* primer mix, 8.5 μl H_2_O, and 1 μl DNA template. The reactions were carried out on a CFX96 Real-Time Detection System (Bio-Rad, Hercules, CA, United States) at 65°C for 30 cycles with each cycle lasting 1 min. Amplification signals were detected by the fluorescent dye included in the reaction, and amplification with a sigmoidal-shaped curve was considered positive. For the standard LAMP, the reaction contained 12.5 μl of WarmStart Colorimetric LAMP 2 × Master Mix with UDG (New England Biolabs, Ipswich, MA, United States), 2.5 μl 10 × *caf1A* primer mix, 9 μl H_2_O, and 1 μl DNA template. The reactions were incubated on a 0.2 ml PCR tube-block (Thermo Fisher Scientific, Pittsburgh, PA, United States) within an enclosed Corning™ LSE™ Digital Dry Bath (Thermo Fisher Scientific, Pittsburgh, PA, United States) at 65°C for 30 min. The presence of the targeted gene was signified by visual observation with naked eyes of color change from red (cresol red as the pH indicator dye) to yellow, whereas the color remained unchanged in the absence of amplification. Positive was defined when a complete color change in a LAMP reaction was observed within 30 min of incubation at 65°C. Distilled water was always included as the negative control for both systems.

### Optimization of Reaction Temperature by Real-Time Loop-Mediated Isothermal Amplification

Real-time LAMP was performed using the DNA of *Y. pestis* strain CO96-3188 (1 pg) for optimization of the reaction temperature. The reactions were carried out on a CFX96 Real-Time Detection System (Bio-Rad, Hercules, CA, United States) at a temperature gradient of 60–70°C (60, 60.7, 62, 64, 66.4, 68.4, 69.5, and 70°C) for 30 cycles, with each cycle lasting 1 min. Four replicates were tested for each temperature. *Ct* values were converted to TTR (time to result) for comparison purposes. A temperature with lower TTR and all four replicates amplified was considered an optimized temperature.

### Cutoff of Reaction Time and Sensitivity Assessment Using a Dry Bath Heater

The DNA of *Y. pestis* strain CO96-3188 of 11 different concentrations (100, 50, 10, 5, 1, 0.5, 0.4, 0.3, 0.2, 0.1, and 0.05 pg) was used to optimize the cutoff time of the LAMP reaction using a dry bath heater. Sensitivity when using the dry bath heater was assessed simultaneously in this step. Limit of detection (LOD) was used for estimating sensitivity. Four replicates were tested for each concentration. LAMP reactions were loaded on a preheated 0.2 ml PCR tube-block and incubated in a Corning™ LSE™ Digital Dry Bath (Thermo Fisher Scientific, Pittsburgh, PA, United States) at 65°C based on the optimization results of reaction temperatures by the real-time LAMP. A color change of the reactions was initially checked after 20 min of incubation and monitored every additional 5 min until there was no more color change observed in the reactions containing tested DNA or until negative controls started to show some level of color change. A longer time with a complete color change in all four replicates of a lower DNA concentration or no color change in all four replicates of negative control was considered the cutoff of the reaction time, and the DNA concentration was considered the LOD using the dry bath heater.

### Sensitivity Assessment by Real-Time Loop-Mediated Isothermal Amplification

The DNA of *Y. pestis* strain CO96-3188 at concentrations of 100, 50, 10, 5, 1, 0.5, 0.1, 0.05, 0.04, 0.03, 0.02, 0.01, and 0.005 pg was tested using real-time LAMP. Reactions were carried out on a CFX96 Real-Time Detection System (Bio-Rad, Hercules, CA, United States) at 65°C for 30 one-minute cycles based on the optimization results for reaction temperature and cutoff of reaction time from the above sections. Four replicates of each concentration were tested. The lower concentration with all four replicates amplified or no amplification in all four replicates of negative control was considered the LOD using real-time LAMP.

### Specificity Assessment

Specificity of the LAMP assay was assessed by testing DNA from genetically close bacterial species within the genus of *Yersinia*, including 10 strains of *Y. pseudotuberculosis* and 2 strains of *Y. enterocolitica*. In addition, a wide variety of other non-*Yersinia* bacterial species (Table 3) was also tested. The reason to include these microorganisms is because some of them share a similar transmission route with *Yersinia* species, either by fleas or other blood-feeding vectors (Brook et al., 2015; Bai et al., 2017; Pawelczyk et al., 2019) or by consumption of contaminated water (Casanovas-Massana et al., 2018). High nucleic acid concentrations (100 pg) with four replicates of each sample were tested. LAMP reactions were carried out on both a dry bath heater and real-time PCR instrument at 65°C for 30 min (which were the optimization results from the above section). A negative result was defined when targeted DNA was not detected in any of the four replicates for each sample.

### Evaluation of the Loop-Mediated Isothermal Amplification Assay

The liver and spleen of rodents are two tissue types commonly tested for *Y. pestis* infection. To evaluate the performance of the LAMP assay, DNA was extracted from the liver and spleen collected from ten 2–4-month-old outbred female CD-1 white mice (Charles River Laboratories, Wilmington, MA, United States) and was first screened for the absence of *Y. pestis* DNA using a pentaplex real-time PCR (Bai et al., 2020). All tissue DNAs were then spiked with the DNA of *Y. pestis* strain CO96-3188 with a concentration of 1 pg and tested using both real-time LAMP and dry bath incubation at 65°C for 30 min to check if any interference would occur from the mouse tissue DNA. Furthermore, one liver DNA sample and one spleen DNA sample collected from the same mouse were randomly picked for sensitivity testing and to compare with pure *Y. pestis* DNA. The final DNA concentrations of *Y. pestis* spiked in the mouse DNA matched the sensitivity testing using the pure DNA of *Y. pestis* strain CO96-3188. To improve quantification, real-time LAMP was conducted for this step. Four replicates of each concentration were tested.

### Testing *Yersinia pestis* Strains With Different Plasmid Profiles

DNAs extracted from 11 *Y. pestis* strains were tested using both the standard LAMP and the real-time LAMP. Among them, three strains possess all three plasmids; five strains lack the pCD1 plasmid; two strains lack the pCD1 and pPCP1 plasmids; and the last one (strain PB6) lacks the pMT1 and pPCP1 plasmids (Table 1). The final DNA concentration of *Y. pestis* was 1 pg in the LAMP reactions, and four replicates of each template were tested.

### Application of the Assay for Testing Field-Collected Fleas

To evaluate the utility of the LAMP assay, we tested 32 fleas (30 *Oropsylla hirsuta* and 2 *Oropsylla tuberculata*) that were collected from prairie dog burrows after plague activities (Enscore et al., 2021) using both the standard LAMP and the real-time LAMP. These fleas had been tested previously using a pentaplex real-time PCR, and two of the samples were reported positive for *Y. pestis* DNA while all others were negative (Bai et al., 2020). The results from the LAMP assay were compared with those from the pentaplex real-time PCR.

## Results

### Optimization of Reaction Temperature by Real-Time Loop-Mediated Isothermal Amplification

**FIGURE 1:**
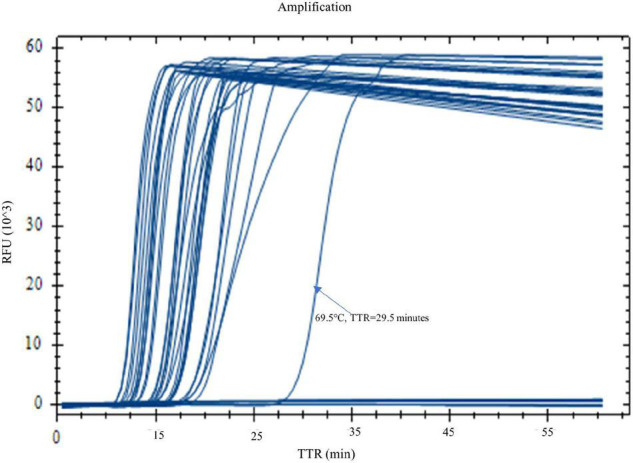
Optimization of reaction temperature by real-time LAMP. LAMP reactions with *Yersinia pestis* DNA (1 pg) were carried out on a real-time PCR instrument at a temperature gradient of 60–70°C (60, 60.7, 62, 64, 66.4, 68.4, 69.5, and 70°C). Four replicates were tested for each temperature. Four out of four replicates were amplified at all temperatures, except for 70°C. Temperatures 66.4 and 64°C generated the best results with shorter TTR and a more even amplification curve.

When the real-time LAMP is run at a temperature gradient of 60–70°C (60, 60.7, 62, 64, 66.4, 68.4, 69.5, and 70°C), all four replicates of *Y. pestis* DNA in the LAMP reactions were amplified at all temperatures except for 70°C (Figure 1 and Table 4). The TTR differed among the temperatures, ranging from 12.4 to 19.6 min (Table 4). The least TTR was at 66.4 and 64°C (12.4 and 12.8 min, respectively). At 64 and 66.4°C, the LAMP reactions not only had shorter TTR but also amplified more consistently (smaller standard deviation) compared to other temperatures (Table 4). Inconsistent amplification (larger standard deviation) was observed at lower or higher temperature; for example, at 69.5°C, one of the four replicates was amplified at about 30 min (Figure 1). Based on the results, 65°C was selected as the optimized reaction temperature and was applied for all subsequent experiments in this work.

**TABLE 4:** Time to result (mean of the TTRs of four replicates) at different temperatures.

Temperature	# Replicates	# Positive	TTR ± SD* (min)
70°C	2	4	N/A
69.5°C	4	4	15.9 ± 1.3
68.4°C	4	4	13.4 ± 2.3
66.4°C	4	4	12.4 ± 0.8
64°C	4	4	12.8 ± 0.8
62°C	4	4	15.7 ± 2
60.7°C	4	4	17.1 ± 0.6
60°C	4	4	19.6 ± 1.2

*SD, standard deviation.

### Cutoff of Reaction Time and Sensitivity Assessment Using the Dry Bath Heater

**FIGURE 2:**
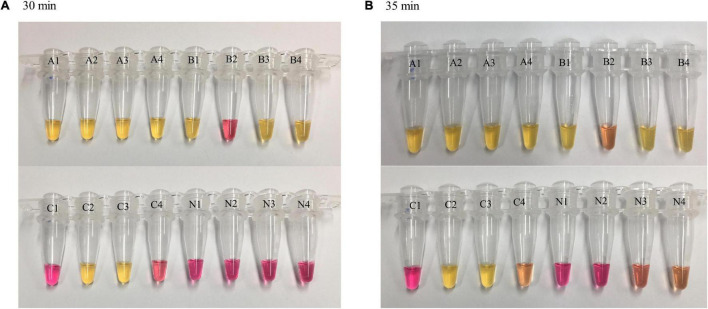
Loop-mediated isothermal amplification reaction time and visual observations of color change. LAMP reactions with different concentrations of *Y. pestis* DNA were carried out on a dry bath heater. Four replicates were tested for each concentration and negative control. At 30 min **(A)**, color change was observed in four of four replicates of reactions with a DNA concentration of 0.2 pg (as well as higher concentration, not shown here) and in partial replicates of reactions with lower DNA concentrations (0.1 and 0.05 pg). Negative control remained unchanged; at 35 min **(B)**, a minor color change was observed in negative control (N3 and N4). A = 0.2 pg, B = 0.1 pg, C = 0.05 pg, N = negative control.

A color change from red (negative) to yellow (positive) was observed at different time points for the LAMP reactions with varying DNA concentrations. The higher the DNA concentration, the shorter the TTR for color change. At 20 min, a complete color change was observed in all four replicates of reactions with DNA concentrations of 100 and 50 pg while reactions with lower concentrations and negative control remained unchanged. At 25 min, a complete color change was observed in all four replicates of reactions with DNA concentrations of 10, 5, 1, and 0.5 pg while reactions with lower concentrations and negative control remained unchanged. At 30 min, a complete color change was observed in all four replicates of reactions with DNA concentrations of 0.4, 0.3, and 0.2 pg, and the negative control remained unchanged, while reactions with lower DNA concentrations showed color change in partial replicates, with 3/4 and 2/4 replicates for DNA concentrations of 0.1 and 0.05 pg, respectively (Figure 2A). The LAMP reactions with these two DNA concentrations were considered negative based on our criteria requiring that all four replicates be called positive. At 35 min, the color change for the DNA concentrations of 0.1 and 0.05 pg was similar to that at 30 min, with some incomplete color change observed (Figure 2B, tubes B2 and C4), and the two DNA concentrations still were considered negative. However, at 35 min, two of four replicates of the negative control started to show some minor color change (Figure 2B, tubes N3 and N4), suggesting that some non-specific reactivity occurred. This was an indication to stop the incubation. In the comparison of the results between 30 and 35 min, 30 min was determined as the cutoff of LAMP reaction time using the dry bath heater. Simultaneously, the LOD was identified as 0.2 pg by visual detection using the dry bath heater.

### Sensitivity and Limit of Detection Identification by Real-Time Loop-Mediated Isothermal Amplification

**FIGURE 3:**
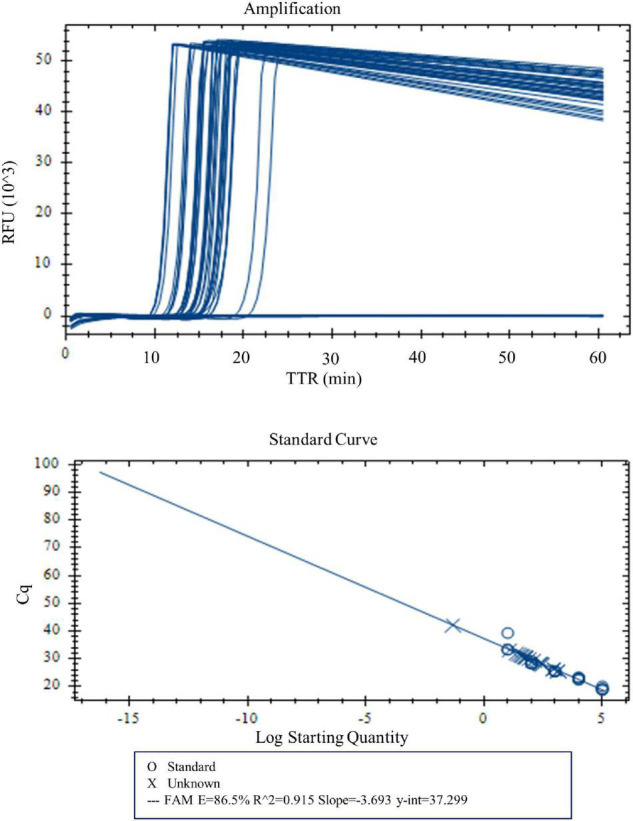
Amplification and standard curve with DNA concentrations 100, 50, 10, 5, 1, 0.5, 0.1, 0.05, 0.04, 0.03, 0.02, 0.01, and 0.005 pg. Four replicates were tested for each concentration. Four of four replicates were amplified for DNA concentrations 100–0.03 pg. TTR was negatively correlated to the DNA concentrations.

With real-time LAMP, the reactions with a DNA concentration of 100–0.03 pg were amplified by all four replicates. The TTR, ranging 10.1 min for 100 pg to 16.2 min for 0.03 pg, is negatively correlated with the DNA concentrations (Figure 3 and Table 5). For lower DNA concentrations, there were 3/4, 3/4, and 1/4 replicate(s) amplified for 0.02, 0.01, and 0.005 pg, respectively, which was considered a negative based on our criteria. Therefore, 0.03 pg was determined as the LOD for the LAMP assay when using the real-time PCR instrument.

**TABLE 5:** Time to result (mean of the TTRs of four replicates) for different concentrations of *Y. pestis* DNA.

DNA concentration	Replicates	# Pos	TTR ± SD* (min)
100 pg	4	4	10.1 ± 0.3
10 pg	4	4	11.9 ± 0.2
1 pg	4	4	13.2 ± 0.1
0.5 pg	4	4	13.5 ± 0.2
0.1 pg	4	4	14.8 ± 0.2
0.05 pg	4	4	15.3 ± 0.8
0.04 pg	4	4	16.0 ± 0.8
0.03 pg	4	4	16.2 ± 0.8
0.02 pg	4	3	N/A
0.01 pg	4	3	N/A
0.005 pg	4	1	N/A

*SD, standard deviation.

### Specificity Assessment

Testing a high DNA concentration (100 pg) of *Y. pseudotuberculosis*, *Y. enterocolitica*, and 20 other non-*Yersinia* bacterial species using the LAMP assay showed no amplification of the targeted gene in any of these microorganisms by either real-time LAMP or dry bath incubation (Table 3), showing 100% specificity of the assay.

### Evaluation of the Loop-Mediated Isothermal Amplification Assay

DNA extracted from mouse spleen and liver spiked with *Y. pestis* DNA (1 pg) all produced amplification within 30 min with both dry bath incubation and real-time LAMP. The average TTR by real-time LAMP was very similar between the two types of *Y. pestis* DNA-spiked tissue with 16.7 and 17.1 min for liver DNA and spleen DNA, respectively (*t* = 2.02, *p* = 0.27), but was significantly longer than that of pure *Y. pestis* DNA (13.2 min, *t* = 1.68, *p* < 0.01).

The LOD was identified as 0.05 pg for both *Y. pestis*-spiked liver DNA and spleen DNA using real-time LAMP. This was lower than the LOD of pure *Y. pestis* DNA (0.03 pg). The average TTR was similar between *Y. pestis*-spiked liver DNA and spleen DNA, with 18.2 and 21.8 min, respectively (*t* = 3.18, *p* = 0.12), but was significantly longer than that of pure *Y. pestis* DNA of the same concentration (15.3 min, *t* = 1.94, *p* < 0.01). These results suggest that the mouse tissue presented some interference with the *Y. pestis* DNA when performing the LAMP assay.

### Testing *Yersinia pestis* Strains With Different Plasmid Profiles

When *Y. pestis* strains with different plasmid profiles were tested using the LAMP assay, all strains that possess the pMT1 plasmid were detected by both real-time LAMP and dry bath incubation within 30 min, although these strains differ in other plasmids; one strain (strain PB6) was not detected with either real-time LAMP or dry bath incubation. This strain, PB6, lacks the pMT1 plasmid (Table 2), and the negative result was expected.

### Application of the Assay for Testing Field-Collected Fleas

**FIGURE 4:**
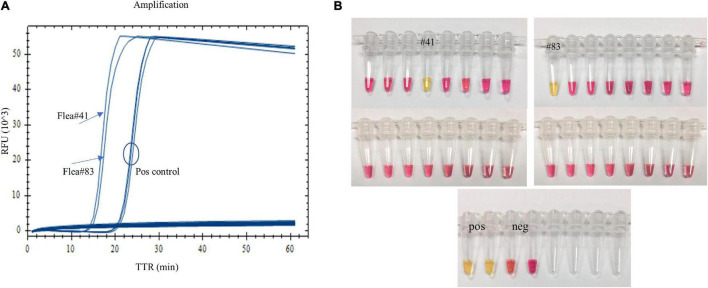
Testing field-collected fleas using the LAMP assay. One *Oropsylla hirsuta* flea (#41) and one *O. tuberculata* flea (#83) previously tested positive were amplified with real-time LAMP **(A)**, and a color change was observed with dry bath incubation **(B)**.

When fleas collected from prairie dog burrows were tested after plague activities using the LAMP assay, *Y. pestis* DNA was detected in one *O. hirsuta* flea (#41) and one *O. tuberculata* flea (#83) with both real-time LAMP and dry bath incubation (Figure 4). The two fleas had been reported *Y. pestis* positive previously by using a pentaplex real-time PCR assay (Bai et al., 2020). The LAMP testing results were identical to the pentaplex real-time PCR testing.

## Discussion

In this work, we developed a LAMP assay targeting the *caf1A* gene for *Y. pestis* detection. As a subunit of the *caf1* operon, *caf1A* plays an important role in capsular biogenesis (Galyov et al., 1990; Karlyshev et al., 1992; Runco et al., 2008; Sebbane et al., 2009; Dubnovitsky et al., 2010). Situated on the pMT1 plasmid, the highly specific *caf1* has been a common target used in developing PCR assays for *Y. pestis* detection (Norkina et al., 1994; Tomaso et al., 2003; Stewart et al., 2008; Singh et al., 2020). Although retaining the same specificity as *caf1*, *caf1A* has not been explored much as a target for *Y. pestis* detection. To our knowledge, this is the first LAMP assay that targets *caf1A* for *Y. pestis* detection.

An ideal LAMP assay uses six of three pairs of primers, which include two outer primers, two inner primers, and two loop primers in the reaction. However, the LAMP primer design is very challenging due to the requirement of the selection of eight separate regions of target nucleic acid sequences, restriction on positioning, and other conditions. It is extremely difficult to design paired loop primers for *Y. pestis* LAMP assays when targeting *caf1*. In the previously reported *Y. pestis* LAMP assays, only one assay included two paired loop primers (Singh et al., 2020), while other assays used only one single loop primer in their assay development (Nunes et al., 2014; Randriantseheno et al., 2020), which possibly was because the primers failed to generate. Using *caf1A*, we were able to generate paired loop primers, which gave us a total of six *caf1A*-specific primers (Table 1) for our LAMP assay development. Although a LAMP assay can work without loop primers, the technology was far too slow for most practical applications in this manifestation, while the addition of loop primers in conjunction with the other primers will significantly increase the speed (Nagamine et al., 2002; Nunes et al., 2014). As a result, our LAMP assay can detect *Y. pestis* in less than 30 min. This faster completion time greatly expedites results compared to other assays which run between 45 and 60 min using four or five primers (Nunes et al., 2014; Feng et al., 2018; Randriantseheno et al., 2020; Singh et al., 2020).

Sensitivity assessment showed that the LOD of our LAMP assay was 0.2 pg of DNA when performed on the dry bath heater where positive results are visually detected by a color change. In the previous *caf1* LAMP assays, Singh et al. (2020) reported the LOD at 0.5 pg; the other two assays reported a much higher LOD, 10 and 3.79 pg, respectively (Nunes et al., 2014; Randriantseheno et al., 2020). Noticeably, Singh et al. included one pair of loop primers in their assay while the other two included one single loop primer. Although different reagents and/or equipment used in each laboratory may have some impacts on the results, the comparisons suggest that inclusion of one pair of loop primers can significantly increase the sensitivity of a LAMP assay. Our LAMP assay is even more sensitive when performed on a real-time PCR instrument with an LOD of 0.03 pg. This was lower than that found by a pentaplex real-time PCR assay that had an LOD of 0.05 pg for *caf1* (Bai et al., 2020). These results may have suggested that *caf1A* is more sensitive than *caf1* in *Y. pestis* detection. Although the LOD of the LAMP assay was low, it still needs further validation for its biological relevance. Nevertheless, because of the extremely high sensitivity, a LAMP assay may risk generating some false amplification if overamplified (Chou et al., 2011). Using the dry bath heater, we observed some minor color change in the negative control when incubation time reached 35 min (Figure 2B). This suggested that the LAMP reaction must be stopped at an earlier time point, and we have set 30 min as the cutoff at which there was no color change for any replicates of the negative control (Figure 2A). One should take extreme caution when using a LAMP assay and make sure that a stringent reaction time cutoff is maintained during testing. Surprisingly, a cutoff time for the LAMP reaction was not determined in many other studies, which may have risked yielding false-positive results. Interestingly, the reaction time seems to have had less of an impact when performing real-time LAMP, from which we did not observe amplifications for the negative control with longer time. On the other hand, if an incomplete color change was observed within 30 min of incubation, intermediate might be considered. Such a result may suggest the targeted DNA is at a very low concentration. An electrophoresis gel may be run to check the presence of the amplicons. Alternatively, visualizing the LAMP products under UV lights may get clearer results as previous LAMP assays did (Nunes et al., 2014; Singh et al., 2020). Although some minor color change in the negative control was observed at 35 min when using the dry bath heater, testing of *Y. pseudotuberculosis*, *Y. enterocolitica*, and a variety of other bacterial species using both real-time PCR instrument and the dry bath heater has demonstrated that the LAMP assay has a 100% specificity. This again suggested having a cutoff time for the LAMP reaction is critical.

Amplification time was negatively related to the DNA concentration in the LAMP reactions. In the setup with visual detection, if the DNA concentration is high enough, the assay may take only a few minutes before color change is observed. A LAMP assay can be stopped at any time if the targeted DNA is detected before the assay completes. This is another advantage of LAMP assay over conventional PCR. Nevertheless, a full 30 min incubation should be accomplished when testing field samples.

Inhibition is often a problem when applying a molecular method for pathogen detection, which sometimes causes a false-negative detection (Schrader et al., 2012). The inhibiting substances may present in the analyzed samples and may affect the sensitivity of the assay (Kaneko et al., 2007; Nkouawa et al., 2010). A previous study reported inhibition from mouse liver, spleen, and lung DNA when testing for the presence of *Y. pestis* using LAMP assay and PCR (Feng et al., 2018). In the current study, we also observed some interference from mouse tissue, which would decrease the sensitivity and increase the time needed for amplification. A strategy to remove the inhibiting effects may be adapted for better consequences (Schrader et al., 2012). This highlights the need to evaluate assay performance when testing alternative samples types.

Using the LAMP assay, we tested 10 *Y. pestis* strains presenting different plasmid profiles. Strains that possess the pMT1 plasmid were all detected. Other plasmids, present or absent, do not impact the detection of the assay. One strain lacking the pMT1 plasmid was not detected as expected. Over time, *Y. pestis* strains lacking certain plasmids have been reported infrequently (Butler, 1972; Meka-Mechenko, 2003). To successfully detect different variants and to elucidate the plasmid profile in a particular strain, multi-target LAMP assay(s) would be required.

We applied the assay to test field-collected fleas. The DNA from these fleas had been tested previously using a pentaplex PCR assay (Bai et al., 2020). The results from the LAMP assay were identical to those using the pentaplex real-time PCR, suggesting the utility of the assay for field investigations.

The quality of DNA differs largely when using different extraction methods. DNA samples used in the current work were prepared using commercial kits, e.g., Qiagen kits, but these might not be available in resource-poor settings. Instead, a low-tech solution, such as boiling preparation, may have to be used. One should be aware that the performance of the LAMP assay may be affected by the DNA quality.

In conclusion, we have developed a highly sensitive, specific, and rapid LAMP assay targeting *caf1A* for rapid detection of *Y. pestis*. The assay can be performed by simply using a dry bath heater and completes within 30 min by incubation at 65°C. The results can be detected by a visual observation-based color change of the reaction. This feature would make the assay extremely useful in regions with limited resources or during field investigations where immediate access to an equipped facility may not be available. The rapidity of the assay is extremely important for medical management, control, and surveillance of plague. The assay also can be performed using a real-time PCR instrument with higher sensitivity and is recommended when quantitative analysis is favored.

## Data Availability Statement

The original contributions presented in the study are included in the article/supplementary material, further inquiries can be directed to the corresponding author.

## Ethics Statement

The animal study was reviewed and approved by Animal Care and Use Committee at the Division of Vector-Borne Diseases, Centers for Disease Control and Prevention.

## Author Contributions

YB conceived, designed, and performed the experiment, analyzed the data, and wrote the manuscript. MR, CP, and SM performed the experiment and reviewed the manuscript. RE supervised, reviewed, and edited the manuscript. All authors contributed to the article and approved the submitted version.

## Author Disclaimer

The conclusion, findings, and opinions expressed by authors contributing to this journal do not necessarily reflect the official position of the United States Department of Health and Human Services, the Public Health Service, the Centers for Disease Control and Prevention, or the authors’ affiliated institutions.

## Conflict of Interest

The authors declare that the research was conducted in the absence of any commercial or financial relationships that could be construed as a potential conflict of interest.

## Publisher’s Note

All claims expressed in this article are solely those of the authors and do not necessarily represent those of their affiliated organizations, or those of the publisher, the editors and the reviewers. Any product that may be evaluated in this article, or claim that may be made by its manufacturer, is not guaranteed or endorsed by the publisher.

## Footnotes

1
http://primerexplorer.jp/
